# Effects of mineralocorticoid receptor antagonists in patients with hypertension and diabetes mellitus: a systematic review and meta-analysis

**DOI:** 10.1038/jhh.2015.119

**Published:** 2015-12-17

**Authors:** S Takahashi, J Katada, H Daida, F Kitamura, K Yokoyama

**Affiliations:** 1 Department of Epidemiology and Environmental Health, Juntendo University Graduate School of Medicine, Tokyo, Japan; 2 Medical Affairs, Pfizer Japan Inc., Tokyo, Japan; 3 Department of Cardiovascular Medicine, Juntendo University Graduate School of Medicine, Tokyo, Japan

## Abstract

Blood pressure (BP) control is important to ameliorate cardiovascular events in patients with diabetes mellitus (DM). However, achieving the target BP with a single drug is often difficult. The objective of this study was to evaluate the antihypertensive effects of mineralocorticoid receptor antagonists (MRAs) as add-on therapy to renin–angiotensin system (RAS) inhibitor(s) in patients with hypertension and DM. Studies were searched through October 2014 in MEDLINE, Embase and the Cochrane Central Register of Controlled Trials. Randomized, controlled trials or prospective, observational studies regarding concomitant administration of MRA and RAS inhibitor(s) in patients with DM were included. Articles were excluded if the mean systolic BP (SBP) was <130 mm Hg before randomization for interventional studies or at baseline for prospective cohort studies. We identified nine eligible studies (486 patients): five randomized placebo-controlled trials; three randomized active drug-controlled trials; and one single-arm observational study. The mean differences in office SBP and diastolic BP (DBP) between the MRA and placebo groups were −9.4 (95% confidence interval (CI) −12.9 to −5.9) and −3.8 (95% CI, −5.5 to −2.2) mm Hg, respectively. Subgroup analysis results for study type, age, baseline office SBP and follow-up duration were similar to those of the main analysis. MRA mildly increased serum potassium (0.4 mEq l^−1^; 95% CI, 0.3–0.5 mEq l^−1^). A consistent reduction of albuminuria across these studies was also demonstrated. MRA further reduced SBP and DBP in patients with hypertension and DM already taking RAS inhibitors. Serum potassium levels should be monitored to prevent hyperkalemia.

## Introduction

Hypertension and diabetes mellitus (DM), which commonly co-exist,^1, 2^ are both established risk factors for cardiovascular-related morbidity and mortality. When both are present, the risk for cerebrovascular disease and coronary artery disease significantly increases.^3^ With intensive reduction in blood pressure (BP) in patients with DM, cardiovascular events, especially stroke, occur less often.^4^ Therefore, strict BP control is important to reduce the cardiovascular risk in patients with DM.

Because they reportedly protect renal function,^5^ renin–angiotensin system (RAS) inhibitors, such as angiotensin-converting enzyme (ACE) inhibitors or angiotensin receptor blockers (ARB), are recommended as first-line antihypertensive therapy for DM.^6, 7^ However, BP control using monotherapy is often difficult in patients with DM; treatment with multiple drugs with different mechanisms for BP reduction is necessary.^8^

Aldosterone is a mineralocorticoid and final product of the renin–angiotensin–aldosterone system. Aldosterone blockade by selective and non-selective mineralocorticoid receptor antagonists (MRAs), such as spironolactone or eplerenone, lowers BP and improves renal function.^9, 10^ MRAs might also be effective in patients with resistant hypertension already treated with ⩾3 antihypertensive medications, including RAS inhibitors.^11^ Because aldosterone production is largely dependent on regulation by the upstream factor angiotensin II, it is possible that RAS inhibitors might, at least partly, attenuate the BP-lowering effects of MRAs due to a reduction in the angiotensin II-dependent production of aldosterone. However, many patients experience the ‘aldosterone breakthrough' phenomenon, which is characterized by serum aldosterone levels returning to or exceeding baseline levels after the initiation of pharmacological blockade of the RAS.^12^ Therefore, MRAs might be effective in patients already treated with RAS inhibitors. However, both RAS inhibitors and MRAs can increase serum potassium levels. Their concomitant use could further increase the risk of hyperkalemia, especially in patients with reduced renal function, including patients with DM. However, the effect of combination MRA and RAS inhibitor treatment on BP and hyperkalemia risk in patients with DM has not been assessed in a large population.

This systematic review and meta-analysis aimed to assess the antihypertensive effect and safety, indicated by serum potassium levels, of MRAs and RAS inhibitors used in combination to treat hypertensive patients with DM.

## Materials and methods

### Search strategy

We performed this systematic review and meta-analysis based on the Cochrane handbook^13^ and Preferred Reporting Items for Systematic Reviews and Meta-Analyses (PRISMA) statement. The following electronic databases were searched: MEDLINE (1946 to 21 September 2014), Ovid MEDLINE(R) In-Process & Other Non-Indexed Citations (29 September 2014), Embase (1974 to 29 September 2014) and the Cochrane Central Register of Controlled Trials (CENTRAL; all dates to 1 October 2014). We used the following search terms: hypertension, hypertensive, blood pressure, diabetes, diabetic, eplerenone and spironolactone. The search was restricted to English articles of human studies; review articles were excluded. Reference lists of retrieved articles were also reviewed.

### Eligibility criteria

Clinical studies regarding the concomitant administration of MRA with RAS inhibitors in patients with type 1 or type 2 DM were included. Prospective observational trials and randomized controlled trials with either parallel groups or a crossover design comparing MRA with placebo or other antihypertensive drugs were included. Articles were excluded if the subjects were aged <18 years, the observation period was <1 month, or mean systolic BP (SBP) was <130 mm Hg before randomization for interventional studies or at baseline for prospective cohort studies.

### Data extraction

A data extraction sheet was developed based on the Cochrane Consumers and Communication Review Group's data extraction template, which was modified for the purpose of this systematic review. The following information was extracted: (1) trial participant characteristics and inclusion criteria; (2) intervention, including MRA type, MRA dose and MRA duration; and (3) outcome measures including SBP, diastolic BP (DBP), serum potassium levels, serum creatinine levels, estimated glomerular filtration rate (eGFR), urinary albumin creatinine ratio (UACR) or urinary protein creatinine ratio (UPCR), and adverse events. The published articles were used for data extraction and raw data were not collected. Assessment of study eligibility was performed independently by two reviewers. Disagreements were resolved by consensus.

### Assessment of methodological quality

Study quality was evaluated according to the Cochrane Handbook. To evaluate potential bias, two reviewers independently assessed the reliability by determining the adequacy of randomization and concealment of allocation, blinding of participants and personnel, blinding of outcome assessments, and extent of loss to follow-up and selective reporting.

### Summary measures

The primary outcome measure was the seated or supine office SBP at the end of the MRA treatment period. The mean difference (MD) in SBP between baseline and post treatment was assessed for prospective cohort studies. Meta-analyses were conducted for DBP, serum potassium, eGFR and adverse events if the values were available in >3 studies.

### Statistical analysis

The pooled effect of the MD between the MRA and placebo groups and 95% confidence intervals (CIs) for each meta-analysis were calculated using the weighted effects of individual studies; forest plots were also produced. Sensitivity analysis was conducted by excluding one study at a time from the original pooled analysis to examine whether any studies had a substantial impact on the model. The *a priori* decision to use a random-effects model for the meta-analyses was based on its assumption that the true effect size varies between studies due to clinical and methodological diversity; it is also more robust than a fixed-effects model, which assumes that the true effect size is the same for all studies. To evaluate the heterogeneity, the fixed-effects model results were used as reference. The Q statistic, which is a statistical test of heterogeneity, was considered significant at *P*⩽0.10. The *I*^2^ statistic was calculated for each analysis to evaluate the percentage of observed variance attributed to between-study heterogeneity rather than chance. Values are reported as mean±s.d., unless otherwise specified. Statistical analyses were conducted using EZR software, version 3.0.2 (Saitama Medical Center, Jichi Medical University, Saitama, Japan), which is a graphical user interface for R (The R Foundation for Statistical Computing, Vienna, Austria).

### Additional analyses

Planned subgroup analyses were undertaken to evaluate the robustness of the meta-analysis for the primary outcome (SBP) with the following categories, when ⩾2 studies had the category: (1) type of trial (parallel or crossover), (2) MRA (eplerenone or spironolactone), (3) mean age at randomization (<65, ⩾65 years), (4) mean SBP at randomization (<150 mm Hg, ⩾150 mm Hg) and (5) length of follow-up (<6 months, ⩾6 months). Bonferroni correction was used for the *P-*values of the subgroup analyses. If the selected number of studies was ⩾10, the possibility of publication bias was to be assessed by evaluating the funnel plots for the MDs for asymmetry.

## Results

### Search strategy results

**Figure 1:**
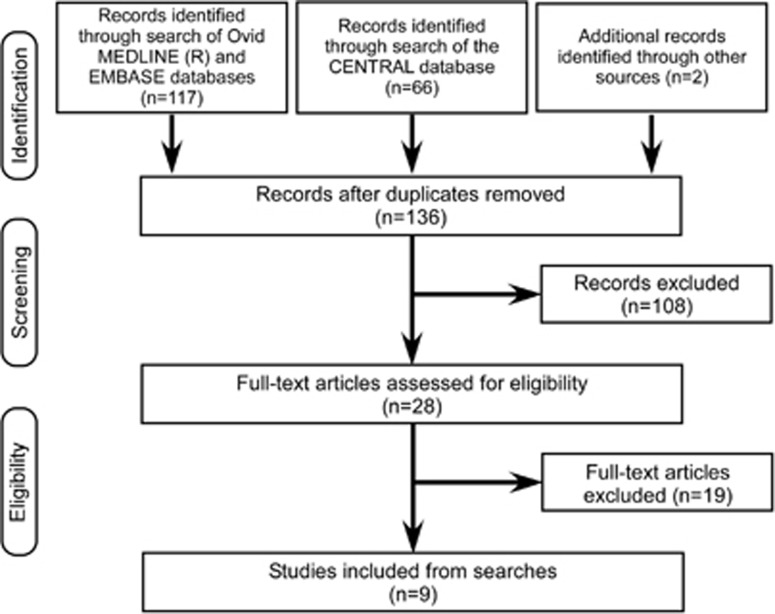
Flow diagram of the search for and inclusion of studies.

The search of the Medline, EMBASE and CENTRAL databases resulted in 185 citations including two studies found by reviewing the reference lists of retrieved articles (Figure 1). Of the 136 studies that remained after removing duplicates, 108 studies were excluded based on abstract review because they did not meet the criteria. Review of the full text resulted in the exclusion of an additional 19 studies that did not meet the inclusion criteria or have enough information to judge if they met the criteria. Therefore, nine studies (486 patients) were included in the analyses.

### Study characteristics

The nine studies consisted of two randomized placebo-controlled trials,^14, 15^ three crossover placebo-controlled trials,^16, 17, 18^ three randomized active drug-controlled trials^19, 20, 21^ and one open-label single-arm prospective cohort study^22^ (Table 1). All studies, except two,^14, 15^ were from a single centre. Five placebo-controlled trials were included in the meta-analysis. Because the randomized active drug-controlled studies had different active drugs as the controls, they were only reviewed in a descriptive manner.

Only patients with type 2 DM and patients with both type 1 and type 2 DM were included in eight studies and one study, respectively. All of the studies used 25 mg per day or 25–50 mg per day of spironolactone as the MRA, and no study used eplerenone. The mean ages of the participants were 53–65 years and were similar in all of the studies. The majority of the participants were men. In the eight studies that reported a baseline SBP, it varied from 134.2±16.5 to 162.7±17.2 mm Hg. Seven of the nine studies targeted patients with albuminuria or proteinuria.

### Intervention

The duration of the intervention varied from 1 month to 18 months in the nine studies (Table 1) and from 1 month to 12 months in the five studies included in the meta-analyses.^14, 15, 16, 17, 18^

### Outcome variables

All selected studies reported office BP.^14, 15, 16, 17, 18, 19, 20, 21, 22^ Of the placebo-controlled studies, no more than two studies reported renal function markers, including eGFR, UACR or UPCR. Therefore, meta-analyses were not conducted for these variables. All of the studies evaluated adverse effects, including the change in serum potassium levels. The one study that did not report serum potassium at the end of the MRA treatment period^17^ was not included in the meta-analysis for serum potassium.

### Quality of study methods

The quality of the studies varied (Table 2). Of the randomized studies, the generation of the random sequence was adequate in only three studies^14, 15, 21^ and unclear in the remaining studies. Allocation concealment was described in only one study^14^ and was unclear in the remaining studies. All five placebo-controlled studies were double blind; three active drug-controlled studies were open-label. Five studies^14, 15, 16, 18, 20^ used an automatic device for BP measurement, which was considered low risk for the introduction of bias in the outcome assessment. One study used a standard mercury sphygmomanometer to measure BP, and three studies had no description of BP measurement devices. Among the eight randomized studies, only one study was analysed using an intention-to-treat basis.^14^ The rate of dropouts ranged from 0 to 30%. Because <10 studies were selected, tests for funnel plot asymmetry were not used to assess publication bias as recommended by the Cochrane handbook.

### Meta-analysis: RAS inhibitors plus MRAs versus RAS inhibitors plus placebo

**Figure 2:**
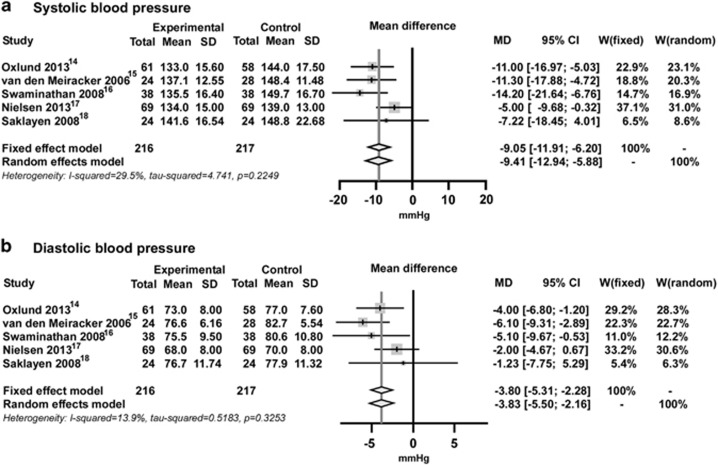
Effect of mineralocorticoid receptor antagonists versus placebo for (**a**) systolic blood pressure and (**b**) diastolic blood pressure. CI, confidence interval; MD, mean difference; W, weight.

The reduction in office SBP with MRA in the parallel and crossover studies was greater than in the placebo-controlled studies (MD=−9.4 mm Hg, 95% CI, −12.9 to −5.9 mm Hg; Figure 2a). The Q-test for SBP was not significant, and the I^2^ value for SBP did not indicate substantial heterogeneity (29.5%, *P*=0.2249). The MD of office DBP was also significant (−3.8 mm Hg, 95% CI, −5.5 to −2.2 mm Hg). There was also no substantial heterogeneity in DBP (Figure 2b).

### Subgroup analyses

The subgroup analyses for type of trial, mean age at randomization, mean SBP at randomization and length of follow-up did not result in different SBP-lowering effects of MRA between the groups, when compared with the main analysis (Table 3). As all the studies used spironolactone, a subgroup analysis with type of MRA was not conducted. The sensitivity analyses found that no study, when removed individually, changed the statistical significance of the pooled result.

### RAS inhibitors plus MRAs versus RAS inhibitors plus other antihypertensive drugs

In the three studies identified as randomized active drug-controlled trials, two studies used diuretics (furosemide^19^ or trichlormethiazide^20^), and one study used an ARB (losartan^21^) as the active drug. In all, three active drug-controlled studies, SBP and DBP in both the study and comparator groups were reduced to a similar extent, without significant between-group differences.

### Furosemide versus MRAs

In the study that compared furosemide and MRA, SBP and DBP decreased to a similar extent in both groups.^19^ The SBP/DBP values at the randomization point for MRA and furosemide were 144±8/76±6 and 141±9/78±6 mm Hg, respectively. At study end, the values were 127±5/72±8 and 138±7/76±3 mm Hg, respectively.

In the study that compared trichlormethiazide and MRA, SBP decreased significantly from baseline in both groups; the between-group difference was not significant (spironolactone: −12±12 mm Hg; trichlormethiazide: −10±13 mm Hg, between-group *P*=0.786).^20^ DBP decreased significantly in the MRA group but not in the trichlormethiazide group; the between-group difference was not significant (spironolactone: −7±13 mm Hg; trichlormethiazide, −3±7 mm Hg, between-group *P*=0.469).

In the study that compared ACE inhibitor/ARB with MRA/ARB, all patients were treated using combined enalapril and losartan at study initiation; this treatment continued in the ACE inhibitor/ARB group.^21^ In the MRA/ARB group, enalapril was replaced with spironolactone after a 2-week washout period. The reduction in both SBP and DBP was significantly greater in the MRA/ARB group than in the ACE inhibitor/ARB group after 18 months (both, *P*<0.001).

### Open label, single arm study of MRAs plus angiotensin-converting enzyme inhibitors and/or angiotensin receptor blockers

In the prospective cohort study, SBP significantly decreased from 141.2±3.5 to 132.5±3.6 mm Hg (*P*=0.002).^22^ A change in DBP was not reported.

### Renal function

All studies recruited diabetic patients with either albuminuria or proteinuria except two; one had approximately half the sample as patients with albuminuria,^14^ and the other did not report whether it included patients with albuminuria.^16^

MRA, but not placebo, induced a marked reduction in UACR, UPCR or urinary albumin excretion rate (UAER) in the studies that reported these values (Table 4). Although UACR did not significantly decrease in the furosemide or RAS inhibitor co-administered group in the active drug-controlled studies, UACR significantly decreased from baseline in the trichlormethiazide group; the change was not significant compared with that in the MRA group.^20^ UACR significantly decreased from baseline (*P*=0.003) in the prospective cohort study.^22^

All studies that reported baseline or change in eGFR values reported decreased values in the MRA group, either significantly or non-significantly. In the placebo groups, the decrease in eGFR was relatively small compared with the MRA group, or eGFR slightly increased.^18^ A placebo-controlled study reported that the reduction in eGFR in the MRA group was largest during the first 3 months after treatment initiation, but remained at the same level afterwards, and the slope of eGFR in the MRA group was similar to that seen in the placebo group.^15^ The reduction in eGFR in the MRA group tended to be larger in patients with a higher baseline eGFR. An active drug-controlled study reported that eGFR significantly decreased from baseline in both the MRA and trichlormethiazide groups; the between-group difference was not significant.^20^ Another active drug-controlled study also reported that eGFR decreased in both the MRA/ARB and ACE inhibitor/ARB groups, without a significant between-group difference.^21^

### Serum potassium and hyperkalemia

**Figure 3:**
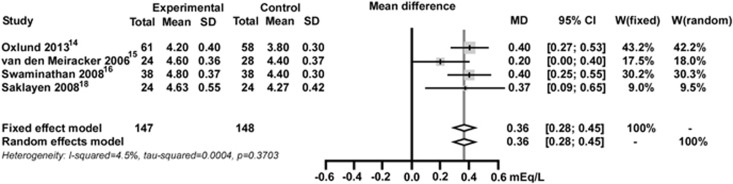
Effect of mineralocorticoid receptor antagonists versus placebo for serum potassium. CI, confidence interval; MD, mean difference; W, weight.

After exclusion of the study that did not report serum potassium,^17^ four of the five placebo-controlled studies (233 patients) were included in the meta-analysis for serum potassium (Figure 3).^14, 15, 16, 18^ The increase in serum potassium at the end of MRA treatment was significantly greater than with placebo (MD=0.36 mEq l^−1^; 95% CI, 0.28–0.45 mEq l^−1^). There was no significant heterogeneity between studies (*I*^2^=4.5%, *P*=0.3703).

Hyperkalemia (serum K+ >5.5 mEq l^−1^) occurred in 16 of the 233 patients (MRA, *n*=13; placebo, *n*=3), and eight discontinued the study drug to adverse events (MRA, *n*=7; placebo, *n*=1; Table 5). Because these studies, except one,^14^ did not include the discontinued subjects in their analyses, serum potassium values were not reported; therefore, these patients were not included in the meta-analysis.

Of the nine studies in this systematic review, hyperkalemia occurred disproportionately more frequently in the MRA group in the study by van den Meiracker *et al.*^15^ Within 2–12 weeks after drug initiation, six patients (MRA, *n*=5; placebo, *n*=1) developed hyperkalemia (serum potassium, 6.0 mmol l^−1^; range, 5.6–6.7 mmol l^−1^) and were excluded from the analysis. The patients with hyperkalemia were older (mean (range): 62.3 (58–71) versus 51.1 (29–78) years) and had higher creatinine (geometric mean (interquartile range): 162 (123–250) versus 91 (77–117) μmol l^−1^), higher serum potassium (4.7±0.3 versus 4.2±0.3 mmol l^−1^), lower eGFR (mean (range): 40 (25–55) versus 74 (58–90) ml per min·1.73 m^2^) and longer DM durations (mean (range): 19.5 (7–35) versus 13.0 (7–39) years) than patients who did not develop hyperkalemia.^15^

Of the 160 patients in the three active drug-controlled studies,^19, 20, 21^ MRA was discontinued in three patients because of asymptomatic hyperkalemia;^21^ no patient developed hyperkalemia in the active drug-controlled groups. After MRA discontinuation, the potassium levels of all three patients returned to normal within 6 months. There were no significant changes in serum potassium during the single-arm prospective cohort study.^22^

## Discussion

This systematic review and meta-analysis aimed to evaluate the antihypertensive effects of MRAs as add-on therapy to RAS inhibitor(s) in patients with hypertension and DM. The most important findings were that the addition of MRA to RAS inhibitor(s) induces a significant additional reduction in SBP and DBP and reduces urinary albumin excretion, whereas marginally elevating plasma potassium concentrations.

Overall, the evidence is sufficiently robust regarding the comparative effectiveness of MRA on blood pressure reduction in hypertensive patients with DM who are already on RAS inhibitors. Although hypertension in patients with DM is often difficult to control, MRA might be a useful antihypertensive therapy. The anti-albuminuric or anti-proteinuric effects of MRA were consistently observed in placebo- and active drug-controlled randomized studies and prospective cohort studies. A potential explanation for the effectiveness of MRA in patients already taking RAS inhibitors includes the other factors, namely elevated potassium, cortisol and adrenocorticotropic hormone levels, that are involved in the stimulation of aldosterone production in the adrenal cortex, in addition to angiotensin II, which is considered the major stimulator. Therefore, aldosterone breakthrough (increase in aldosterone after reduction) could occur even when RAS inhibitors, such as ACE inhibitors and ARBs, are consistently effective. Second, with high salt intake, renin production is suppressed, and BP is not effectively controlled by RAS inhibitors. However, aldosterone- and mineralocorticoid receptor (MR)-mediated signaling might be potentiated, and MR-dependent mechanisms might be involved in the aetiology of hypertension,^23^ providing another explanation for the effectiveness of MRAs on BP, combined with RAS inhibition.

All publications used for the meta-analysis report the effects of spironolactone, not eplerenone, a less potent but more selective MRA compared with spironolactone. Both spironolactone and eplerenone are competitive antagonists of MR with very similar molecular structures, and it is not likely that their antihypertensive mechanisms are different. It is reasonable to assume that similar anti-hypertensive effects would be obtained when eplerenone is used at appropriate doses instead of spironolactone. However, the effect of MR blockade on the metabolic profile may vary by the MRA used due to differences in selectivity. Eplerenone has been shown to improve insulin resistance in obese, diabetic mice.^24^ In contrast, Homma *et al.*^25^ demonstrated that spironolactone, but not eplerenone, negatively affected parameters of glucose metabolism such as blood glucose in a rat model of metabolic syndrome, likely mediated through increased aldosterone levels. Similarly, results from the CHARM study have indicated that spironolactone use may be associated with an increased risk for the development of diabetes.^26^ As described in the review article of Vaidya *et al.*,^27^ dysregulated aldosterone physiology is associated with early cardio-metabolic abnormalities. Taken together, it is plausible that eplerenone might be superior to spironolactone when used for treatment of hypertensive patients with diabetes and glucose intolerance. Further investigation is necessary.

As a potential mechanism for BP reduction by MRAs, it is widely acknowledged that multiple mechanisms are involved other than the diuretic action of MRAs, including improved smooth muscle cell and endothelium function^28^ and the attenuation of enhanced sympathetic drive^29^ through the inhibition of the renal aldosterone-MR system or central nervous system. For example, the administration of 25–50 mg per day eplerenone, a selective MRA, did not have a clear diuretic effect but still showed significant nighttime BP reduction.^30^ Therefore, MRA could also be effective for BP lowering when added to a diuretic. In addition, obesity or visceral fat accumulation in some patients with DM might contribute to the BP reduction by MRA. An adipose tissue-derived factor might be associated with the enhanced generation of aldosterone by adrenal cortex cells, and aldosterone is reportedly involved in adipocyte dysfunction.^31^ Abnormal adipocytokine production might directly and indirectly contribute to high BP.

A consistent reduction of UACR and/or UPCR with MRA treatment was also demonstrated. MRA might slow the progression of renal dysfunction in hypertensive patients with decreased renal function. Although not included in this systematic review because essential hypertensive patients were not the target population, a previous study demonstrated that eplerenone reduced albuminuria in patients with type 2 DM.^32^ Because albuminuria or proteinuria is not only a symptom of renal injuries, but also an aggravating factor of chronic kidney disease, a reduction in albuminuria might stabilize or improve renal dysfunction. Diabetic nephropathy is a very common and serious complication in patients with DM, often leading to haemodialysis. Therefore, a renoprotective effect of MRA could provide clinical benefits to hypertensive patients with DM in addition to its BP-lowering effects.

The present systematic review also suggested that eGFR is uniformly decreased while renal function is markedly improved, based on UACR or UPCR, with MRA treatment. Similar effects are also observed after the initiation of RAS inhibitor treatment, which might improve the long-term prognosis of renal function.^5^ The mechanisms underlying the reduction in eGFR by MRA are not clear. A plausible explanation is the relief of hyperfiltration, which is thought to frequently occur in the kidneys with DM nephropathy and might burden the residual functioning glomeruli.^33^

Increased plasma potassium levels are the most serious concern in patients receiving MRA treatment. The concomitant use of RAS inhibitors and decreased renal function are considered risk factors for hyperkalemia, or increased plasma potassium levels, with MRA treatment. However, the extent of the increase in plasma potassium levels shown in the current meta-analysis is not large, even in hypertensive patients with DM with concomitant MRA and RAS inhibitor use, and is similar to that reported in essential hypertension patients.^34^ Therefore, the increase in plasma potassium is within a predictable and manageable range for most patients. Nonetheless, it is noteworthy that there were more patients that developed hyperkalemia during MRA treatment than in those receiving placebo. In clinical practice, the risk/benefit balance should be considered with MRA add-on treatment in patients with hypertension and DM taking RAS inhibitors based on the individual risk factors for each patient.

Although gynecomastia reportedly occurs in 6–21% of spironolactone-treated patients,^35, 36^ there was only one incidence of gynecomastia reported in the MRA group^21^ in the nine studies, which might be attributed to the relatively short treatment duration of MRA therapy or the low spironolactone dose (25–50 mg per day) in these patients (Table 1). Eplerenone might be more appropriate than spironolactone for some patients due to the low incidence of gynecomastia because eplerenone is more specific for MR. Eplerenone has an ~370-fold less binding affinity for androgen receptors than spironolactone and, in contrast to spironolactone, does not bind to progesterone and glucocorticoid receptors even at high concentrations.^37^ However, in some countries, eplerenone is contraindicated in hypertensive patients who have both DM and albuminuria.

In this systematic review, we used a SBP of 130 mm Hg as the cut-off point for the inclusion criteria of the studies based on the Japanese Society of Hypertension Guidelines for the Management of Hypertension (JSH 2014).^38^ The target SBP remained unchanged for diabetic patients, even though the society acknowledged hypertension guidelines in Europe^39^ and the United States^40, 41^ were revised from an SBP of 130 mm Hg to an SBP of 140 mm Hg. We maintained an SBP cut-off of 130 mm Hg because the incidence of stroke is still higher in Japan than in Europe and the United States and because there is accumulating evidence regarding a reduced incidence of stroke with an SBP<130 mm Hg. We analysed whether our results would change if we used a cut-off point of 140 mm Hg for SBP in our systematic review and found that if we used the 140 mm Hg cut-off point, only one study^21^ would be excluded, but this would not have changed the results. The excluded study was not a placebo-control study and it would not have been included in the meta-analysis.

The following clinical questions should be addressed in future studies: does eplerenone (a selective MRA) differ from spironolactone (a non-selective MRA) with regard to the risks and benefits for treating hypertension in patients with DM; does the addition of an MRA to RAS inhibitors for hypertension in patients with DM prevent the onset of cardiovascular events, the progression to end-stage renal disease, or the initiation of dialysis? In addition, do any genetic factors contribute to hyperkalemia during MRA treatment?

Our study has several limitations. Seven^16, 17, 18, 19, 20, 21, 22^ out of nine studies included in this systematic review were from single centre. However, these 9 studies included 13 institutions located in 6 countries. Therefore, we believe that the results of this systematic review can be applied to the general population. The majority of studies did not adequately report the study methods to allow the assessment of study quality. Only one study^14^ reported the use of intention-to-treat analysis; the treatment effect might be overestimated in the other trials. The six patients who developed hyperkalemia shortly after drug initiation and were excluded from the analysis^15^ could not be included in the meta-analysis of serum potassium, which might have affected the results. Publication bias was not assessed because the number of studies included in the meta-analysis was <10. Therefore, publication bias might exist.

The results indicate that add-on treatment with an MRA in patients with hypertension and DM who do not achieve SBP<130 mm Hg with an ACE inhibitor and/or an ARB provides further benefits for BP and UACR and/or UAER. Reduced albuminuria or proteinuria might also occur in patients with overt albuminuria or proteinuria. Despite observing only a few cases with a marked increase in serum potassium, careful assessment of the patient's background to identify any risk factors for hyperkalemia is necessary in clinical practice. The risk/benefit balance of add-on MRA therapy should be considered on an individual basis.


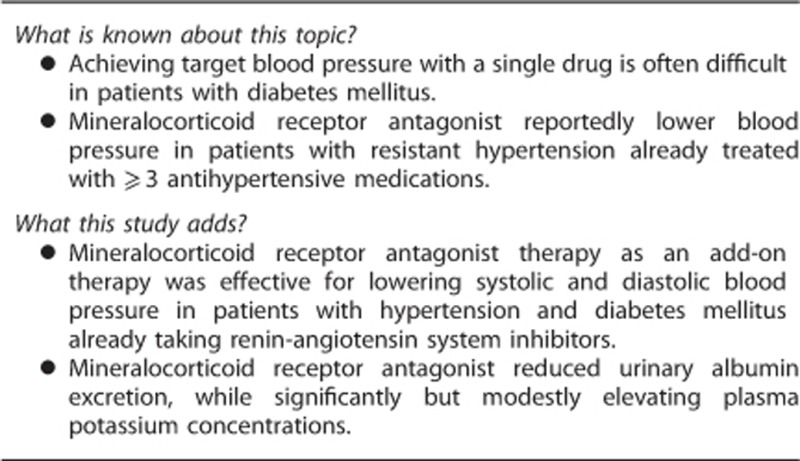


## Figures and Tables

**Table 1:** Characteristics of studies assessing the antihypertensive effect of mineralocorticoid receptor antagonists added to renin–angiotensin system inhibitors in hypertensive patients with diabetes mellitus

*Study*	*Treatment*	*Age, years*	*Male, %*	*Baseline SBP,* *mm Hg*	*Baseline DBP,* *mm Hg*	*Subjects for analysis,* n	*Discontinued rate, %*	*Type of DM*	*Follow-up duration, months*
Oxlund *et al.*^14^	Spironolactone 25–50 mg per day	62.9±7.1	75	144±15	79±11	61	0	T2DM	4
	Placebo	63.9±6.9	78%	139±15	76±8	58	0		
van den Meiracker *et al.*^15^	Spironolactone 25–50 mg per day	55.2 (38–78)^a^	69.6	144±17	80±10	24	17.2	T2DM	12
	Placebo	55.2 (29–75)^a^	58.6	148± 13	82±8	28	6.7		
Swaminathan *et al.*^16^	Spironolactone 25–50 mg per day	62.6 (48–78)^a^	73.7	162.7±17.2	88.9±9.2	38	24	T2DM	1
	Placebo								
Nielsen *et al.*^17^	Spironolactone 25 mg per day	53±11	76.8	NA	NA	69	NA	T1DM (n=46)	
	Placebo			NA	NA			T2DM (*N*=23)	2
Saklayen *et al.*^18^	Spironolactone 25–50 mg per day	64.5	100	153.6±26.0	79.6±12.2	24	20	Unspecified	3
	Placebo			154.5±21.2	79.7±12.9				
Ogawa *et al.*^19^	Spironolactone 25 mg per day	63.5±5.5	NA	144±8	76±6	20	0	T2DM	12
	Furosemide 20 mg per day	61.2±6.4	NA	141±9	78±6	10	0		
Hase *et al.*^20^	Spironolactone 25 mg per day	65±7	66.7	149±20	79±14	18	5.3	T2DM	6
	Trichlormethiazide 2 mg per day	62±9	80	146±10	79±11	15	11.8		
Esteghamati *et al.*^21^	Spironolactone 25 mg per day + Losartan 50–100 mg per day	57.8±8.9^b^	68.9^c^	134.2±16.5	83.1±9.8	52	29.7	T2DM	18
	Enalapril 30–40 mg per day + Losartan 50–100 mg per day	58.3±9.3^b^	64.5^c^	144.1±21.7	86.1±11.0	45	27.4		
Davidson *et al.*^22^	Spironolactone 25 mg per day	60.4±9.2	58	141.2±3.5	NA	24	20.0	T2DM	1

Abbreviations: DBP, diastolic blood pressure; DM, diabetes mellitus; NA, not applicable; SBP, systolic blood pressure; T1DM; type 1 diabetes mellitus; T2DM, type 2 diabetes mellitus.

Data are expressed as mean and s.d., unless otherwise indicated.

a Mean and range.

b Mean ages of subjects at randomization.

c Mean percentage of men at randomization.

**Table 2:** Quality of studies assessing the antihypertensive effect of mineralocorticoid receptor antagonists added to renin–angiotensin system inhibitors in hypertensive patients with diabetes mellitus

*Study*	*Generation of random sequence*	*Concealment of allocation*	*Blinding of participants and personnel*	*Blinding of outcome assessment*	*Incomplete outcome data*	*Selective reporting*
Oxlund *et al.*^14^	Low risk	Low risk	Low risk	Low risk	Low risk	Low risk
van den Meiracker *et al.*^15^	Low risk	Unclear risk	Low risk	Low risk	High risk	Low risk
Swaminathan *et al.*^16^	Unclear risk	Unclear risk	Low risk	Low risk	Low risk	Low risk
Nielsen *et al.*^17^	Unclear risk	Unclear risk	Low risk	Unclear risk	Unclear risk	High risk
Saklayen *et al.*^18^	Unclear risk	Unclear risk	Low risk	Low risk	Low risk	Low risk
Ogawa *et al.*^19^	Unclear risk	Unclear risk	High risk	Unclear risk	Low risk	Low risk
Hase *et al.*^20^	Unclear risk	Unclear risk	High risk	Low risk	Unclear risk	Low risk
Esteghamati *et al.*^21^	Low risk	Unclear risk	High risk	High risk	High risk	Low risk
Davidson *et al.*^22^	NA	NA	NA	Unclear risk	High risk	Low risk

Abbreviation: NA, not applicable.

**Table 3:** Summary of results from pre-specified subanalyses

*Comparison*	*Number of studies*	*Studies included*	*Number of participants*	*RR random effect model (95% CI)*	*I*^*2*^ *result for heterogeneity* (P-value)
*Trial structure*					
Parallel group, placebo controlled	2	Oxlund *et al.*,^14^ van den Meiracker *et al.*^15^	171	−11.14 (−15.56, −6.72)^a^	0% (0.9472)
Crossover, placebo controlled	3	Swaminathan *et al.*,^16^ Nielsen *et al.*,^17^ Saklayen *et al.*^18^	131	−8.49 (−14.65, −2.33)^a^	52.5% (0.1219)
					
*Mean age at randomization*					
⩾65	0		0	NA^b^	
<65	5	Oxlund *et al.*,^14^ van den Meiracker *et al.*,^15^ Swaminathan *et al.*,^16^ Nielsen *et al.*,^17^ Saklayen *et al.*^18^	302	−9.41 (−12.94, −5.88)^a^	29.5% (0.2249)
					
*Mean SBP at randomization*					
⩾150 mm Hg	2	Swaminathan *et al.*,^16^ Saklayen *et al.*^18^	62	−12.03 (−18.36, −5.70)^a^	3% (0.3099)
<150 mm Hg	2	Oxlund *et al.*,^14^ van den Meiracker^15^	171	−11.14 (−15.56, −6.72)^a^	0% (0.9472)
					
*Length of follow-up*					
⩾6 Months	1	van den Meiracker *et al.*^15^	52	NA^b^	
<6 Months	4	Oxlund *et al.*,^14^ Swaminathan *et al.*,^16^ Nielsen *et al.*,^17^ Saklayen *et al.*^18^	250	−9.07 (−13.48, −4.66)^a^	41.4% (0.163)

Abbreviations: CI, confidence interval; NA, not applicable; RR, relative risk.

a Statistically significant.

b Analysis not conducted due to the limited number of studies that included the variable.

**Table 4:** Change in albuminuria or proteinuria following treatment for blood pressure

*Study*	*Treatment group*	*Albuminuria/proteinuria* n *(%)*	*Baseline UACR or UPCR (mg g^−1^ Cr)*	*Post-treatment UACR or UPCR (mg g^−1^ Cr)*	*Change from baseline (mg g^−1^ Cr or %)*	*P-value for change from baseline*	*P-value for difference between groups*
Oxlund *et al.*^14^	Spironolactone 25–50 mg per day	31 (51)	UACR: 27.4 (6.2–2770)^a^	NA	UACR: −7.3 mg/g (−1093 to 12.2)^a^ −26.6%	0.001	0.001
	Placebo	27 (47)	UACR: 32.8 (6–418)^a^	NA	UACR: 0 mg g^−1^ (−74 to 146.3)^a^ 0%	Non-significant	
van den Meiracker *et al.*^15^	Spironolactone 25–50 mg per day	52 (100)	UACR: 571.7 (292.9–953.1)^b^ UPCR: 0.98 (0.61–1.53)^b^	NA	UACR: −44.2% (−64.4 to −24.0)^c^ UPCR: −40.6% (−57.8 to −23.4)^c^	NA	0.002
	Placebo		UACR: 899.1 (386.7–2522.1)^b^ UPCR: 1.28 (0.56–2.32)^b^	NA	UACR: −14.3% (−43.5 to 14.9)^c^ UPCR: −13.5% (−41.8 to 14.9)^c^	NA	
Swaminathan *et al.*^16^	Spironolactone 25–50 mg per day Placebo	NA	NA	NA	NA	NA	NA
Nielsen *et al.*^17^	Spironolactone 25 mg per day	69 (100)	NA	UAER: 433 (295, 636)d,^e^	NA	NA	<0.001
	Placebo		NA	UAER: 605 (411, 890)d,^e^	NA	NA	
Saklayen *et al.*^18^	Spironolactone 25–50 mg per day	24 (100)	UPCR: 1.80 ±1.78^b^	UPCR: 0.79±0.99^b^	−57%	0.004	Non-significant
	Placebo		UPCR: 1.24±1.13^b^	UPCR: 1.57±2.13^b^	24%	0.35	
Ogawa *et al.*^19^	Spironolactone 25 mg per day	30 (100)	UACR: 240±85	UACR: 140±38	−100 mg g^−1^Cr	<0.05	<0.05
	Furosemide 20 mg per day		UACR: 244±70	UACR: 329±103	85 mg g^−1^Cr	Non-significant	
Hase *et al.*^20^	Spironolactone 25mg per day	33 (100)	UACR: 605.6 (362.2–1,012.5)^d^	NA	−57.6%±21.3%	<0.001	0.27
	Trichlormethiazide 2 mg per day		UACR: 582.6 (351.8–946.9)^d^	NA	−48.4%±27.1%	<0.001	
Esteghamati *et al.*^21^	Spironolactone 25 mg per day + Losartan 50–100 mg per day	97 (100)	UAER: 105.0 (62.5, 281.8)e,^f^	UAER: 29.0 (16.5, 69.4)e,^f^	UAER: −60.5 (−148.8, −16.4)e,^c^	<0.001	0.038
	Enalapril 30–40 mg per day + Losartan 50–100 mg per day		UAER: 82.5 (44.3, 340.5)e,^f^	UAER: 105.5 (25.3, 510.8)e,^f^	UAER: 22.0 (−110.3, 108.9)e,^c^	0.809	
Davidson *et al.*^22^	Spironolactone 25 mg per day	24 (100)	UACR: 273.4±40.1	UACR: 194.7±34.1	NA	0.003	NA

Abbreviations: Cr, creatinine; NA, not applicable; UACR, urinary albumin creatinine ratio; UAER, urinary albumin excretion rate; UPCR, urinary protein creatinine ratio.

a Median 5th and 95th percentiles.

b UACR or UPCA values were converted from mg mmol^−1^ to mg g^−1^ (UACR) or g g^−1^ (UPCR) (1 mg g^−1^=1 μg mg^−1^=0.113 mg mmol^−1^).

c 95% CI.

d Geometric mean (95% CI).

e UAER (mg per 24 h).

f Median (interquartile range).

**Table 5:** Adverse events due to treatment for blood pressure

*Study*	*Drugs*	*Hyperkalemia after randomization (K+ >5.5) mEq l^−1^,* n	*Discontinued study due to adverse events* (n)
Oxlund *et al.*^14^	Spironolactone 25–50 mg per day	1	Hyperkalemia (1), symptomatic hypotension (1)
	Placebo	0	
van den Meiracker *et al.*^15^	Spironolactone 25–50 mg per day	11	Hyperkalemia (5)
	Placebo	3	Hyperkalemia (1), hospitalization (1)
Swaminathan *et al.*^16^	Spironolactone 25–50 mg per day	1	Hyperkalemia (1)
	Placebo	0	
Nielsen *et al.*^17^	Spironolactone 25 mg per day	0	
	Placebo	0	
Saklayen *et al.*^18^	Spironolactone 25–50 mg per day	0	
	Placebo	0	
Ogawa *et al.*^19^	Spironolactone 25 mg per day	0	
	Furosemide 20 mg per day	0	
Hase *et al.*^20^	Spironolactone 25mg per day	0	
	Trichlormethiazide 2 mg per day	0	Hyponatremia (1), unspecified drug intolerance (1)
Esteghamati *et al.*^21^	Spironolactone 25 mg per day+Losartan 50–100 mg per day	3	Asymptomatic hyperkalemia (3), bothersome gynecomastia (1)
	Enalapril 30–40 mg per day+Losartan 50–100 mg per day	0	
Davidson *et al.*^22^	Spironolactone 25 mg per day	0	
